# Identification of CT Values That Could Be Predictive of Necrosis (N-CTav) in Hepatocellular Carcinoma after Lenvatinib Treatment

**DOI:** 10.3390/curroncol29050266

**Published:** 2022-05-04

**Authors:** Makoto Chuma, Hideki Yokoo, Atsushi Hiraoka, Kazuhiko Ueda, Takahiro Yokoyama, Kunihiko Tsuji, Noritomo Shimada, Haruki Uojima, Satoshi Kobayashi, Nobuhiro Hattori, Tomomi Okubo, Masanori Atsukawa, Toru Ishikawa, Koichi Takaguchi, Akemi Tsutsui, Hidenori Toyoda, Toshifumi Tada, Yoshinori Saito, Shunji Hirose, Takaaki Tanaka, Kazuhisa Takeda, Masako Otani, Zenjiro Sekikawa, Tsunamasa Watanabe, Hisashi Hidaka, Manabu Morimoto, Kazushi Numata, Tatehiro Kagawa, Michiie Sakamoto, Takashi Kumada, Shin Maeda

**Affiliations:** 1 Gastroenterological Center, Yokohama City University Medical Center, Yokohama 232-0024, Japan; y.takahiro.0730@icloud.com (T.Y.); kazu1968@yokohama-cu.ac.jp (K.T.); kz_numa@yokohama-cu.ac.jp (K.N.); 2 Department of Surgery, Division of Hepato-Biliary-Pancreatic Surgery and Transplant Surgery, Asahikawa Medical University, Asahikawa 078-8510, Japan; hi-yokoo@mua.biglobe.ne.jp; 3 Gastroenterological Center, Ehime Prefectural Central Hospital, Matsuyama 790-0024, Japan; hirage@gmail.com (A.H.); tanaka_ladg@yahoo.co.jp (T.T.); 4 Diagnostic Imaging Center, The Cancer Institute Hospital of Japanese Foundation for Cancer Research, Tokyo 135-8550, Japan; kazuhiko.ueda@jfcr.or.jp; 5 Center of Gastroenterology, Teine Keijinkai Hospital, Sapporo 006-8555, Japan; ktsuj@keijinkai.or.jp (K.T.); takashi.kumada@gmail.com (T.K.); 6 Division of Gastroenterology and Hepatology, Otakanomori Hospital, Kashiwa 277-0863, Japan; norimos@jcom.home.ne.jp; 7 Department of Gastroenterology, Internal Medicine, Kitasato University School of Medicine, Sagamihara 252-0375, Japan; kiruha@kitasato-u.ac.jp (H.U.); hisashi7@kitasato-u.ac.jp (H.H.); 8 Department of Hepatobiliary and Pancreatic Medical Oncology, Kanagawa Cancer Center Hospital, Yokohama 241-8585, Japan; kobayashis@kcch.jp (S.K.); m-morimoto@kcch.jp (M.M.); 9 Department of Internal Medicine, Division of Gastroenterology and Hepatology, St. Marianna University School of Medicine, Kawasaki 216-8511, Japan; n2hattori@marianna-u.ac.jp (N.H.); twatanab@marianna-u.ac.jp (T.W.); 10 Division of Gastroenterology, Nippon Medical School, Chiba Hokusoh Hospital, Inzai 270-1694, Japan; ma60154@nms.ac.jp; 11 Division of Gastroenterology and Hepatology, Nippon Medical School, Tokyo 113-8603, Japan; momogachi@yahoo.co.jp; 12 Department of Gastroenterology, Saiseikai Niigata Hospital, Niigata 950-1104, Japan; toruishi@ngt.saiseikai.or.jp; 13 Department of Hepatology, Kagawa Prefectural Central Hospital, Takamatsu 760-8557, Japan; k.takaguchi@chp-kagawa.jp (K.T.); amitsu6557@yahoo.co.jp (A.T.); 14 Department of Gastroenterology and Hepatology, Ogaki Municipal Hospital, Ogaki 503-8502, Japan; tkumada@he.mirai.ne.jp; 15 Department of Internal medicine, Japanese Red Cross Himeji Hospital, Himeji 670-8540, Japan; tadat0627@gmail.com; 16 Department of Gastroenterology, Asahikawa-Kosei General Hospital, Asahikawa 078-8211, Japan; yoshi99@poplar.ocn.ne.jp; 17 Department of Internal Medicine, Division of Gastroenterology and Hepatology, Tokai University School of Medicine, Isehara 259-1193, Japan; hs099212@tsc.u-tokai.ac.jp (S.H.); kagawa@tokai.ac.jp (T.K.); 18 Diagnostic Pathology, Yokohama City University Medical Center, Yokohama 232-0024, Japan; motani@yokohama-cu.ac.jp; 19 Diagnostic Radiology, Yokohama City University Medical Center, Yokohama 232-0024, Japan; tauko@yokohama-cu.ac.jp; 20 Department of Pathology, Keio University School of Medicine, Tokyo 160-8582, Japan; msakamot@z5.keio.jp; 21 Faculty of Nursing, Gifu Kyoritsu University, Ogaki 503-0001, Japan; 22 Department of Gastroenterology, Yokohama City University Hospital, Yokohama 236-0004, Japan; smaeda@yokohama-cu.ac.jp

**Keywords:** hepatocellular carcinoma, lenvatinib, molecular targeted agents, complete response, CT value

## Abstract

Purpose: To assess the utility of measurement of the computed tomography (CT) attenuation value (CTav) in predicting tumor necrosis in hepatocellular carcinoma (HCC) patients who achieve a complete response (CR), defined using modified Response Evaluation Criteria in Solid Tumors (mRECIST), after lenvatinib treatment. Method: We compared CTav in arterial phase CT images with postoperative histopathology in four patients who underwent HCC resection after lenvatinib treatment, to determine CTav thresholds indicative of histological necrosis (N-CTav). Next, we confirmed the accuracy of the determined N-CTav in 15 cases with histopathologically proven necrosis in surgical specimens. Furthermore, the percentage of the tumor with N-CTav, i.e., the N-CTav occupancy rate, assessed using Image J software in 30 tumors in 12 patients with CR out of 571 HCC patients treated with lenvatinib, and its correlation with local recurrence following CR were examined. Results: Receiver operating characteristic (ROC) curve analysis revealed an optimal cut-off value of CTav of 30.2 HU, with 90.0% specificity and 65.0% sensitivity in discriminating between pathologically identified necrosis and degeneration, with a CTav of less than 30.2 HU indicating necrosis after lenvatinib treatment (N30-CTav). Furthermore, the optimal cut-off value of 30.6% for the N30-CTav occupancy rate by ROC analysis was a significant indicator of local recurrence following CR with 76.9% specificity and sensitivity (area under the ROC curve; 0.939), with the CR group with high N30-CTav occupancy (≥30.6%) after lenvatinib treatment showing significantly lower local recurrence (8.3% at 1 year) compared with the low (<30.6%) N30-CTav group (*p* < 0.001, 61.5% at 1 year). Conclusion: The cut-off value of 30.2 HU for CTav (N30-CTav) might be appropriate for identifying post-lenvatinib necrosis in HCC, and an N30-CTav occupancy rate of >30.6% might be a predictor of maintenance of CR. Use of these indicators have the potential to impact systemic chemotherapy for HCC.

## 1. Introduction

Hepatocellular carcinoma (HCC) is the fourth leading cause of cancer-related deaths globally [1]. The systemic therapy of HCC has changed remarkably in the past few decades, with the introduction of novel molecular targeted agents (MTAs), such as lenvatinib, leading to improved patient progression-free survival [2,3,4,5,6,7,8,9]. It has been clinically shown that MTAs targeting vascular endothelial growth factor (VEGF) and other molecules can significantly reduce tumor blood flow on CT images [3,10]. The modified response evaluation criteria in solid tumors (mRECIST) have been developed to overcome the limitations of standard RECIST criteria in assessing the response to therapy of HCC, including evaluation of tumor blood flow [11,12]. Currently, mRECIST has become the standard tool for measurement of radiological endpoints in the early/intermediate stages of HCC [13], while current guidelines recommend both RECIST and mRECIST for assessment of advanced tumor stages [13]. The mRECIST have been proven to capture higher objective response rates in tumors treated with molecular therapies, and these responses have been shown to be independently associated with better survival [14,15].

Although evaluation using mRECIST is an excellent tool, it is not clear whether the site of decreased “early enhancement” in the tumor (suggestive of a complete response (CR) or partial response (PR) by mRECIST evaluation) after MTA treatment is pathologically necrotic or viable. Moreover, the future course of HCC patients with CR according to mRECIST following MTA therapy is uncertain. In terms of continuing MTA treatment or predicting the course of treatment, it is clinically important to determine whether the area in the HCC with a CR after MTA treatment according to mRECIST pathologically consists of necrotic or viable tissue. Furthermore, it is significant to determine the criteria that might predict subsequent tumor progression following a diagnosis of CR with MTA therapy. Although a few previous studies have used CT attenuation values (CTav), defined as the radiodensity of the tissue being assessed, to evaluate HCC viability and necrosis after transarterial chemoembolization (TACE) [16,17], there are so far no reports on such evaluations using CTav after MTA treatment.

In this study, we attempted to identify CTav that can be used to identify necrosis in HCC tumors after lenvatinib treatment (N-CTav), and to clarify the association between the tumor N-CTav occupancy rate and maintenance of CR following lenvatinib treatment.

## 2. Materials and Methods

### 2.1. Study Design and Patients

This retrospective, multicenter study included unresectable HCC (u-HCC) patients who were treated with orally administered lenvatinib (Lenvima®; Eisai Co., Ltd., Tokyo, Japan) from April 2018 to October 2020. The initial dose of lenvatinib was 12 mg/day for those weighing over 60 kg and 8 mg/day for those weighing less than 60 kg. We identified 608 patients with u-HCC treated with lenvatinib during the observation period. Of the 608 cases, we evaluated the records of 571 patients in whom the treatment response was evaluated properly and who had adequate clinical data. Of the 571 cases, four cases who underwent hepatectomy after lenvatinib treatment at Asahikawa Medical University [*n* = 2] and Yokohama City Medical Center [*n* = 2] were analyzed separately. The baseline characteristics of these patients are shown in Supplementary Table S1. Furthermore, of the 571 cases, a total of 15 patients achieved complete response (CR), from among whom 12 patients in whom CT scans were taken before and after treatment at ten institutions in Japan (Teine Keijinkai Hospital [*n* = 2], Otakanomori Hospital [*n* = 2], Kitasato University Hospital [*n* = 1], St. Marianna University School of Medicine Hospital [*n* = 1], Nippon Medical School [*n* = 1], Ehime Prefectural Central Hospital [*n* = 1], Kanagawa Cancer Center [*n* = 1], Tokushima Prefectural Central Hospital [*n* = 1], Saiseikai Niigata Hospital [*n* = 1], and Yokohama City Medical Center [*n* = 1]) were enrolled in this study.

### 2.2. CT Imaging

The imaging methods used were based on general CT imaging methods used for the diagnosis of HCC [7,13]. Quadruple-phase helical CT (i.e., unenhanced, hepatic arterial, portal venous, and equilibrium phases) was performed using a helical scanner (HiSpeed Advantage; GE Medical Systems, Milwaukee, WI, USA). Multidetector (MD) CT was performed before contrast medium administration and during the hepatic arterial, hepatic venous and delayed phases. The scanning parameters were tube current of 200–400 mA (automatic tube current modulation), section collimation of 5 mm, and table speed of 5 mm/s during a single-breath hold for helical acquisition of 25 to 30 s depending on the liver size. Images were obtained in a craniocaudal direction and were reconstructed every 5 mm to provide contiguous sections. All patients received 1.6 mL/kg total body weight of an intravenous nonionic contrast medium containing an iodine concentration of 560 mg/mL (520–600 mg/mL) (Iomeron 400; Bracco Imaging or Omnipaque 350; GE Healthcare, Chicago, CA, USA). Automatic bolus-tracking techniques with automated scan triggering software were used. Hepatic arterial phase and venous phase scanning were started automatically at 20 and 60 s, respectively. The delayed phase was started 180 s after the start of contrast material injection. CT was performed using a 5-mm contiguous axial section to encompass the whole liver.

### 2.3. Evaluation of Therapeutic Response

Clinical diagnoses of HCCs were made according to the diagnostic criteria of the European Association for the Study of the Liver (EASL) guidelines [13]. Treatment response was evaluated by enhanced CT after introducing lenvatinib at 4–12 weeks, in accordance with the mRECIST protocol [12]. Lenvatinib was continued until identification of progressive disease (PD) or occurrence of unmanageable adverse events (AEs). We defined local recurrence as disease progression based on mRECIST, i.e., re-vascularization of the area of CR or growth of the lesion previously diagnosed as achieving CR. Local recurrence time was defined as the time period from achievement of a CR with lenvatinib treatment until occurrence of local tumor progression, including hypervascularization of the CR lesion. We analyzed the association between local recurrence time and the CT value.

### 2.4. Image Analysis

All the CT images were initially reviewed using liver window settings (window level, 50–100 Hounsfield unit [HU]; window width, 170 HU) on a 2000 × 2000 picture archiving and communications system (PACS; GE Healthcare Integrated Imaging Solution) monitor, following which the window setting for each patient was adjusted as needed. The region of interest (ROI) was set as a circular or oval-shaped 50 mm^2^ area. The ROIs were drawn electrically, the attenuation value of the ROIs was measured twice on average, and the mean values were obtained. This method was based on previous reports of ROI studies of CT images [16,17].

**Figure 1:**
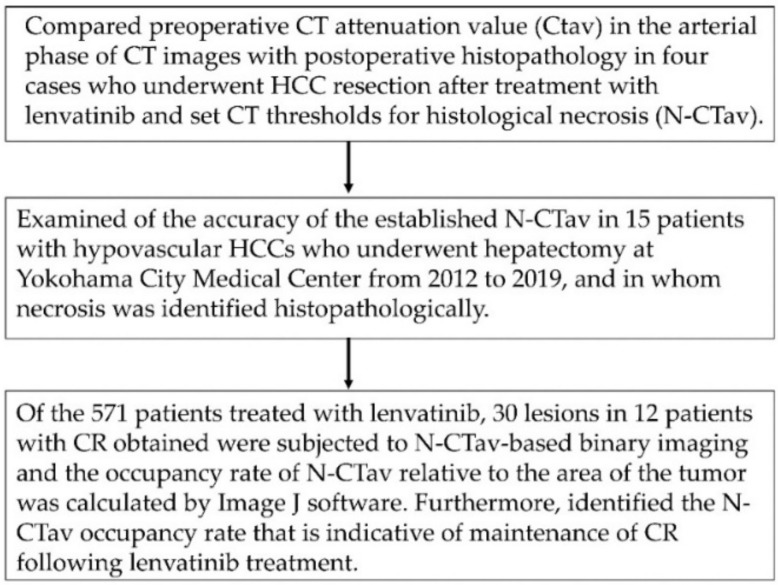
Flow chart showing the study methodology. CT, computed tomography; HCC, hepatocellular carcinoma; Image J software (IJ 1.46r), Image Processing and Analysis in Java software.

The following investigations were conducted to examine the CTav that is indicative of necrosis after lenvatinib administration, and the association between percentage of tumor area occupied by CT values below the set CTav (CTav occupancy rate) and subsequent recurrence. First, we compared preoperative CTav in the arterial phase of CT images with postoperative histopathology in four cases who underwent HCC resection after treatment with lenvatinib, and used the values to set CT thresholds for histological necrosis (N-CTav). Second, we examined the accuracy of the established N-CTav in 15 patients with hypovascular HCCs who underwent hepatectomy at Yokohama City Medical Center during a different time period (from 2012 to 2019), and in whom necrosis was identified histopathologically. Next, binary images based on N-CTav were created for 30 lesions in 12 patients with CR following lenvatinib, and the occupancy rate of N-CTav relative to the maximum area of the tumor was calculated by Image Processing and Analysis in Java (Image J) software (IJ 1.46r) according to the manual available on the Image J official website (Available online: http://rsb.info.nih.gov/ij/docs/guide/index.html) (accessed on 2 October 2012) [18,19]. Briefly, particles were counted and the number of objects in a binarized (black, mode 255 and white, mode 0 binarized) image were measured. Then, we measured the occupancy of N-CTav as the number of black (mode 255) particles/total count. As the primary endpoint, we attempted to establish the N-CTav that predicts necrosis and the N-CTav occupancy rate in binary images that is indicative of maintenance of CR following lenvatinib treatment. A flow diagram of the research is shown in Figure 1. 

### 2.5. Statistical Analysis

Categorical variables were compared using Fisher’s exact test, and continuous variables were evaluated by the Mann-Whitney U test. Data are expressed as the mean ± standard error of the mean (SEM). Significant differences were detected using non-parametric testing. Cumulative local tumor progression was calculated from the day of determination of CR to the date of local tumor progression using the Kaplan-Meier (K-M) method. Differences were evaluated by log-rank testing. Independent factors for local tumor progression were assessed using the Cox proportional hazard regression model. All statistical analyses were carried out using EZR (Saitama Medical Center, Jichi Medical University, Saitama, Japan), which is a graphical user interface for R (The R Foundation for Statistical Computing, Vienna, Austria).

## 3. Results

### 3.1. Characteristics of HCC Patients Who Achieved a Complete Response following Lenvatinib Therapy

The clinical characteristics at baseline, before lenvatinib treatment, of the HCC patients who achieved CR are shown in Table 1. In this study, there were 11 and 1 patients with Child-Pugh (C-P) grade A and B severity, respectively. According to BCLC stage classification, there were nine and three HCC patients with stage B and C disease, respectively. The median observation period was 831 days in 12 patients with HCC.

Additionally, in order to analyze the correlation between pathologically identified necrosis and CTav, 15 separate HCC cases with pathologically identified necrosis in hepatectomy specimens at Yokohama City Medical Center from 2012 to 2019 were evaluated. The baseline characteristics of these patients are shown in Supplementary Table S2.

### 3.2. Preoperative Computed Tomography and Pathological Findings of the Resected Specimens

**Figure 2:**
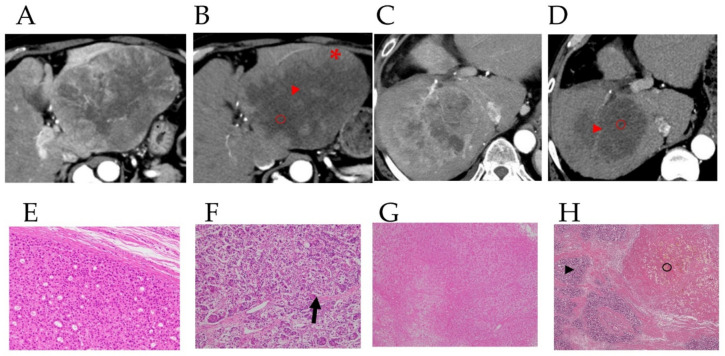

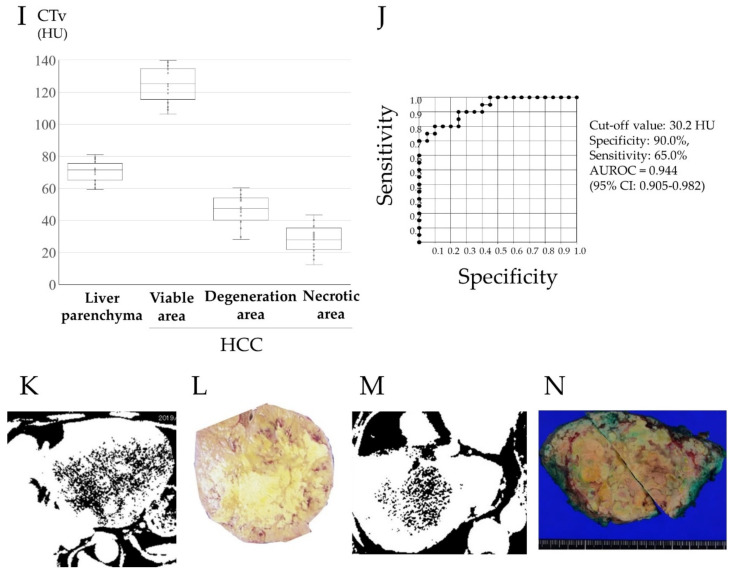
Preoperative contrast-enhanced computed tomography images and pathological findings in the resected specimen. Images of the arterial phase of computed tomography (CT) in HCC patients before (**A**,**C**) and after (**B**,**D**) lenvatinib administration, and pathological findings in hepatectomy specimens from HCC patients after lenvatinib therapy in these patients (**E**–**H**). 2E shows the postoperative histopathological findings corresponding to the asterisk (*) in the CT image in Figure 2B. The arrowheads in Figure 2B,D correspond to the arrowheads in Figure 2F,H, and the circles in Figure 2B,D correspond to Figure 2G, and the circle in Figure 2H. The arrow in Figure F indicates the artery in the degenerated tumor. CT values (CTav) corresponding to the four histological regions (liver parenchyma (LP), viable area of tumor (VA), degenerated area of tumor (DA), and necrotic area of tumor (NA)) (**I**). HU, Hounsfield unit. Receiver-operating characteristic (ROC) curve analysis of CTav for differentiating necrotic areas from degenerated tumor areas (**J**). AUC, area under the ROC curve. Two binary images based on preoperative N30-CTav in HCC patients (**K**,**M**) and the postoperative macroscopic findings corresponding to the two binary images (**L**,**N**). N30-CTav: a CTav of 30 HU was set as the threshold value for histological necrosis.

Figure 2 shows CT images in the arterial phase in two HCC patients before lenvatinib treatment (Figure 2A,C), with attenuated early enhancement after lenvatinib treatment (Figure 2B,D). Figure 2E shows the postoperative histopathological findings corresponding to the asterisk (*) in the CT image in Figure 2B; histopathological images of the areas corresponding to the arrowheads in Figure 2B,D are shown in Figure 2F and the arrowhead in Figure 2; and the areas shown by the circles in Figure 2B,D correspond to the histopathological images shown in Figure 2G and the circle in Figure 2H. Postoperative histopathology showed that the tumor contained several degenerated areas (Figure 2G, and the arrowhead in Figure 2H) along with necrotic areas (Figure 2F, and the circle in Figure 2H) in areas with attenuation of early enhancement (arrowhead and circles in Figure 2B,H) in arterial phase CT images after lenvatinib treatment. In addition, some arteries remained undestroyed in the degenerated tumor (Figure 2F arrow). In these four cases of HCC resection after lenvatinib treatment, the histological specimen was subdivided into four regions: liver parenchyma, viable area of the tumor, degenerated area of the tumor, and necrotic area of the tumor, and the corresponding CTav was measured at five points in each region. The association between CTav on preoperative CT images after lenvatinib treatment and postoperative histopathological findings in the four HCC patients are shown in Figure 2I. The CTav corresponding to the “viable area” in the resected HCC specimen was 125.7 ± 10.9 HU (* in Figure 2B), CTav corresponding to “necrotic areas” in the resected specimen was 27.8 ± 8.4 HU (circle in Figure 2B,D), and the CTav corresponding to the “degeneration area” in the resected specimen was 46.7 ± 9.2 HU (arrowhead in Figure 2B,D). The CTav of normal liver parenchyma was 70.1 ± 6.6 HU (Figure 2I).

ROC curve analysis of the association between CTav and pathological findings, performed to verify the ability of CTav to predict pathological necrosis, revealed an area under the ROC curve (AUC) of 0.925 (95% CI: 0.886–0.964) at the optimal cut-off value of 30.2 HU for CTav, with 90.0% specificity and 65.0% sensitivity for discriminating between necrosis and degeneration as identified histopathologically (Figure 2J). Based on the above finding, we set <30.2 HU as the CTav indicative of tumor necrosis after molecular targeted therapy for HCC on CT images, and termed this value as N30-CTav. Binary images based on N30-CTav were created, and CT images with black fill for areas with CTav < 30 HU are shown in Figure 2K,M, which represent the region close to the necrotic area in the gross image of the resected specimen (Figure 2L,N).

Next, to verify whether the set N30-CTav accurately predicts necrosis, we measured CT values (CTv) in ROIs in pre-hepatectomy CT images of 15 poorly differentiated HCCs (seen as hypovascular tumors in arterial phase CT images) with histopathologically-confirmed tumor necrosis, with the ROIs corresponding to the histopathologically-identified necrotic areas in the hepatectomy specimens. The clinicopathological characteristics of these cases are shown in Supplementary Table S2. The CTv corresponding to “necrotic areas” in the resected specimen was 27.0 ± 8.0 HU, which was significantly lower than the CTv corresponding to “viable areas of poorly differentiated HCC (VA of poorly dHCC)” in the resected specimen of 50.0 ± 10.9 HU (*p* < 0.01, Supplementary Figure S1A). ROC curve analysis revealed an AUC of 0.939 (95% CI: 0.914–0.963) at the optimal cut-off value of 30.4 HU for CTv, with 92.5% specificity and 66.0% sensitivity in discriminating necrotic areas from VAs of poorly differentiated HCC in pathological specimens (Supplementary Figure S1B).

Based on these results, we concluded that 30 HU is a reasonable CTv to identify necrosis after molecular targeted therapy.

### 3.3. Association between N30-CTav Occupancy Rate of the Tumor and Local Tumor Progression

**Figure 3:**
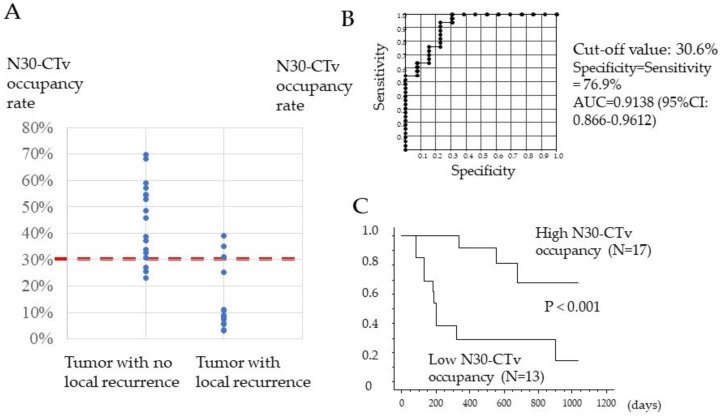
Association between percentage of tumor tissue with CT attenuation values (30 HU) that could be predictive of necrosis (N30-CTav) occupancy and local recurrence-free survival. (**A**) Distribution of N30-CTav occupancy rates between hepatocellular carcinoma (HCC) tumors with local recurrence and HCC tumors with no local recurrence. N30-CTav, threshold CT value (30 HU) for histological necrosis in tumors diagnosed as complete response after lenvatinib treatment. (**B**) Receiver-operating characteristic (ROC) curve analysis of the N30-CTav occupancy rate for predicting local recurrence and no local recurrence. AUC, area under the ROC curve. N30-CTav, a CTav of 30 HU was set as the threshold value for histological necrosis. (**C**) Kaplan-Meier analysis of local tumor progression in 30 tumor lesions with a complete response, stratified by the N30-CTav occupancy rate (grouped by N30-CTav occupancy cut-off values of ≥30.6% and <30.6%).

In the binarized images based on N30-CTav, the proportion of N30-CTav areas relative to the maximum tumor area was examined in 30 tumors in 12 HCC patients who achieved a CR with lenvatinib treatment, using Image J software (IJ 1.46r). Figure 3A shows the N30-CTav occupancy rates in HCC tumors with no local recurrence, which were significantly higher (*p* < 0.001, 44.7%; 23.1–69.8%) than those in HCC tumors with local recurrence (15.0%; 3.2–39.0%). Furthermore, we attempted to establish the N30-CTav occupancy rate in binary images that is indicative of maintenance of CR (no local recurrence) with lenvatinib treatment, using ROC analysis. ROC curve analysis revealed an AUC of 0.9138 (95%CI: 0.866–0.912) at the optimal cut-off value of 30.6% for N30-CTav occupancy, with 76.9% specificity and sensitivity as a significant indicator of CR maintenance (Figure 3B). Furthermore, the high N30-CTav (≥30.6%) occupancy group (N = 17) showed significantly lower local recurrence (1 years/3 years, 8.3%/31.2%) compared with the low N30-CTav (<30.6%) group (N = 13) (1 years/3 years, 61.5%/85.6%) (Figure 3C).

### 3.4. Case Presentations

**Figure 4:**
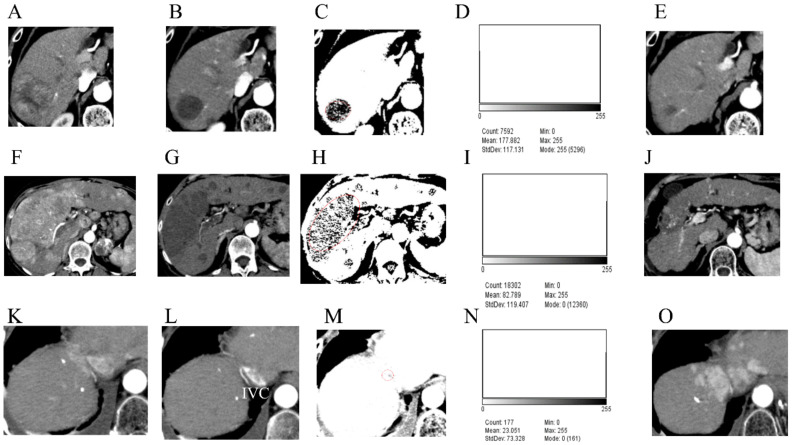
The occupancy rates of CT attenuation values (30 HU) that are predictive of necrosis (N30-CTav) following lenvatinib treatment in three hepatocellular carcinoma (HCC) cases and their subsequent outcomes. Arterial phase of computed tomography (CT) before lenvatinib treatment (**A**,**F**,**K**) and on the day of defining the case as achieving a complete response (CR) after lenvatinib treatment (**B**,**G**,**L**). Binarized images based on N30-CTav on CT images obtained on the day of definition as CR (**C**,**H**,**M**). Histograms showing the occupancy rate of CTav of below 30 HU (N30-CTav) (**D**,**I**,**N**). CT images 1 year (**E**), 2 years (**J**), and 3 months (**O**). after the day of definition as CR. N30-CTav, a CTav of 30 HU was set as the threshold for histological necrosis. Red circles: target tumor area where CR was obtained. IVC, inferior vena cava.

Three cases of HCC with different N30-CTav occupancy rates are shown in Figure 4.

A 72-year-old man presented with HCC predominantly located in the right lobe of the liver (Figure 4A). After 12 weeks of lenvatinib treatment, contrast-enhanced CT showed disappearance of early enhancement in the tumor area, which was evaluated as CR using mRECIST (Figure 4B). Binary images based on N30-CTav are shown in Figure 4C. A histogram of N30-CTav occupancy in the HCC tumor using Image J analysis on the same binary images showed an occupancy rate of 65.9% (Mode 255 count/Total count: 5296/7592 = 0.698) (Figure 4D). CT scan performed one year after the evaluation showed maintenance of CR and marked shrinkage of the tumor (Figure 4E), and since it was determined that the tumor was completely necrotic, lenvatinib administration was discontinued. There has been no evidence of tumor progression since then.

A 68-year-old woman presented with massive, advanced HCC predominantly located in the right lobe of the liver, invading the major branch of the portal vein (Figure 4F). After 12 weeks of treatment with lenvatinib, contrast-enhanced CT showed disappearance of early enhancement in the tumor area, which was evaluated as CR using mRECIST (Figure 4G). Binary images based on N30-CTav are shown in Figure 4H. A histogram of N30-CTav occupancy in the HCC using Image J software analysis on the same binary images indicated an occupancy rate of 32.5% (Total count-Mode 0 count/Total count: ((18,302 − 12,360)/18,302 = 0.325) (Figure 4I). CT scan performed two years after the evaluation showed maintenance of CR and marked shrinkage of the tumor, and since it was determined that the tumor was completely necrotic (Figure 4J), lenvatinib administration was discontinued. There has been no evidence of tumor growth since then.

A 74-year-old man presented with HCC predominantly located in segment 8 of the liver (Figure 4K). After 12 weeks of treatment with lenvatinib, contrast-enhanced CT showed disappearance of early enhancement in the tumor area, which was evaluated as CR using mRECIST (Figure 4L). Binary images based on N30-CTav are shown in Figure 4M. A histogram of N30-CTav occupancy in the HCC using Image J analysis on the same binary images indicated an occupancy rate of only 9.0% (Total count-Mode 0 count/Total count: ((177 − 161)/177 = 0.090) (Figure 4N). CT performed four months after the evaluation showed an increase in tumor size with marked early staining (Figure 4O).

## 4. Discussion

In this study, we determined a CTav of less than 30.2 HU as the threshold value that might be indicative (N30-CTav) of pathological necrosis in HCC tumors after lenvatinib treatment. Moreover, tumors in which more than 30.6% of the maximum tumor area was occupied by a CTav of N30-CTav after lenvatinib treatment showed a low rate of local recurrence. These results are novel and could provide important scientific information for the practice of MTA for HCC.

The systemic therapy for HCC has changed remarkably in the past few decades, and the introduction of novel MTAs, such as lenvatinib, has improved patient progression-free survival [2,3]. Since HCC is a hypervascularized tumor, MTA treatment efficacy is evaluated by both RECIST and mRECIST, which are based on tumor blood flow-contrast effect on CT [10,11,12,13,14,15]. A recent systematic review reported that objective response by mRECIST is an independent predictor of overall survival in patients with advanced HCC [20].

However, no reports have so far determined whether areas of non-enhancement (lesions rated as CR by so-called mRECIST evaluation) on CT after MTA treatment are necrotic. Furthermore, there are no reports evaluating whether tumors defined as achieving CR after MTA treatment using mRECIST subsequently progress. Even in cases evaluated as CR on mRECIST, there is a large discrepancy between necrosis and degeneration in terms of the subsequent course of the disease. It is, hence, clinically very important to distinguish whether HCC tumors that have been evaluated by mRECIST as achieving a CR after MTA are necrotic or degenerated, in order to estimate the patient’s prognosis and the need for continuation of treatment.

Few studies have reported on the evaluation of HCC necrosis after TACE, etc. using CT values [16,17]. The first report defined necrotic tissue as having a CTav of 17.1 HU in the portal venous phase in comparison with hepatic cysts. The second report noted that an iodized-oil defect area (IODA) in HCC treated with TACE was strongly suggestive of viable tumor when the attenuation difference was more than 20 HU on at least one contrast-enhanced phase in quadruple-phase helical CT. The difference in the CT values predictive of necrosis in the present study and those in the previous reports might be due to the different evaluation conditions. In this study, since we evaluated not only resected specimens after molecular targeted therapy, but also the images of histopathological specimens of HCC cases with necrosis, we believe that the CT values determined in this study are more reflective of necrosis.

The difference in CTav between tumor necrosis and degeneration could be explained by the following mechanism. Tumor cell degeneration following MTA is associated with a reduction in the expression of angiogenesis-related molecules, such as VEGF receptors in tumors [21,22,23], leading to a decrease in blood flow in the degenerated tumor and non-enhancement of the degenerated tumor on contrast-CT. In contrast, in areas of necrosis, the blood vessels are completely destroyed and no contrast medium enters the tumor, while in degenerated areas of tumors, the blood vessels are not completely destroyed, and hence, some blood does enter the degenerated area even though the blood flow is severely reduced for the reasons described above (Figure 2F arrow), leading to the higher CTav as compared to necrotic tumors.

The reason why an N30-CTav occupancy rate of more than 30.6% is predictive of a reduced likelihood of local recurrence is that this CTav suggests the presence of necrosis in the tumor, indicating a CR following MTA, and hence, the higher occupancy rate is one of the manifestations of the anti-tumor effect. On the other hand, it is unclear why degenerating tumors sometimes subsequently become more active. Therefore, further studies are needed to confirm whether the local recurrence of HCC following lenvatinib therapy is low in tumors with a post-lenvatinib N30-CTav occupancy rate of more than 30.6%.

The results of this study are clinically valuable since they might enable objective differentiation of whether the HCC tumors have undergone necrosis in response to MTA treatment and might eventually lead to the development of criteria for the follow-up of CR lesions after MTA therapy. At this time, we believe it is important for clinicians to follow up on patients after MTA treatment, as shown by the results of this study.

This study has some limitations, including its retrospective nature. Although this was a multicenter study, the number of patients analyzed was small. Future prospective studies are required to address our findings, evaluating more patients in a multicenter setting using the same protocols. Another limitation is whether the results can be applied to MTA treatment other than lenvatinib. CT values of necrosis in lesions that were assessed as CR due to molecular targeted therapies other than lenvatinib were not investigated in this study. However, since we demonstrated that CT values of 30 HU are suggestive of necrosis in surgical cases, which is the same as that with lenvatinib, it is possible that CT values for other drugs might be similar. In future, it would be desirable to validate N-CTv values in CR lesions by mRECIST following other molecular targeted therapies.

## 5. Conclusions

In conclusion, the N30-CTav determined in this study might be an appropriate cut-off value to identify necrosis in HCCs after MTA treatment, and the N30-CTav occupancy rate of 30.6% might be predictive of maintenance of CR. Use of these indicators might potentially affect systemic chemotherapy for HCC.

## Figures and Tables

**Table 1:** Characteristics of HCC patients who achieved a complete response following lenvatinib therapy.

	*n* = 12
Median age, years (range)	73 (25–84)
Sex (Male/Female)	7/5
Cause of HCC (HBV/HCV/NBNC)	4/6/2
Child-Pugh grade (A/B)	11/1
Child-Pugh score (5/6/7)	8/3/1
mALBI (1/2a/2b)	6/3/3
ECOG PS (0/1)	11/1
BMI (kg/m^2^) (range)	22.0 (19.9–31.8)
Extrahepatic metastasis, *n* (%)	2 (16.7%)
MVI *n* (%)	1 (8.3%)
BCLC stage (B/C)	9/3
TNM stage (II/III/IV)	1/8/3
MTA naïve, *n* (%)	11 (91.7%)
Past history of TACE, *n* (%)	8 (66.7%)
AFP (range)	136 (1.0–1686)
DCP (range)	239 (12–67,900)
Median observation period, days (range)	831 (262–1104)

HBV, hepatitis B virus; HCV, hepatitis C virus; NBNC, non-B, non-C (hepatitis B virus surface antigen-negative/hepatitis C virus antibody-negative); mALBI grade, modified albumin-bilirubin grade; ECOG PS, Eastern Cooperative Oncology Group performance status; BMI, body mass index; MVI, major venous invasion; BCLC, Barcelona clinic liver cancer; TNM stage, tumor node metastasis stage according to the Liver Cancer Study Group of Japan, 6th edition; MTA, molecular targeted agents; TACE, transcatheter arterial chemoembolization; AFP, alpha-fetoprotein; DCP, des-gamma-carboxy prothrombin.

## Data Availability

The data that support the findings of this study are available from the corresponding author upon reasonable request.

## Supplementary Materials

The following supporting information can be downloaded at: https://www.mdpi.com/article/10.3390/curroncol29050266/s1, Table S1: Clinicopathological characteristics of four HCC cases who underwent hepatectomy after lenvatinib treatment, Table S2: Clinicopathological characteristics of 15 HCC cases with histopathologically confirmed necrosis in hepatectomy specimens, Figure S1: CT values (CTa) in necrotic areas and viable areas in poorly differentiated hepatocellular carcinoma (VA of poorly dHCC).

## Abbreviations

HCC, hepatocellular carcinoma; RECIST, Response Evaluation Criteria in Solid Tumors; mRECIST, modified Response Evaluation Criteria in Solid Tumors; OS, overall survival; PFS, progression-free survival; OR, objective response; MTA, molecular targeted agent; AFP, α-fetoprotein; DCP, des-gamma-carboxy prothrombin; TNM, Tumor Node Metastasis classification; BCLC, Barcelona Clinic Liver Cancer, CP; Child-Pugh; mALBI, modified albumin-bilirubin index; TACE, transcatheter arterial chemoembolization.
