# Supplementary material for: Identification of CT Values That Could Be Predictive of Necrosis (N-CTav) in Hepatocellular Carcinoma after Lenvatinib Treatment
Source: Curr Oncol. 2022 May 4;29(5):3259–71. doi: 10.3390/curroncol29050266 (PMC9139739; doi:10.3390/curroncol29050266)
Supplement: Supplementary file 1 [file curroncol-29-00266-s001.zip › curroncol-1613868-supplementary.pdf]

Supplementary Table S1. Clinicopathological characteristics of four HCC cases who underwent hepatectomy after lenvatinib treatment

Variable	n=4
Median age, years	68 (65-76)
Sex (Male/Female), n: %	3/1: 75.0/25.0%
Cause of HCC (HBV/HCV/NBNC), n: %	1/1/2: 25.0/25/0/50.0%
Child-Pugh score 5/6, n: %	2/2: 50.0/50.0%
mALBI (1/2a/2b)	1/2/2
TKI 1st line / 2nd line~	4/0: 100/0%
AFP (ng/mL)	314.5 (7-35300)
DCP (AU/mL)	1354 (8-151000)
Median tumor size (mm)	96 (53-124)
No. of tumor nodules (solitary/multiple)	2/2: 50.0/50.0%
Differentiation (well/moderate/poor)	0/3/1: 0/75/25%
MVI, n (%)	0 (0.0%)
BCLC stage (A/B/C) n: %	0/4/0: 0/100.0/0%
TNM stage (II/III/IV) LCSGJ 6th	2/2/0: 50.0/50.0/0%

HCC, hepatocellular carcinoma; HBV, hepatitis B virus; HCV, hepatitis C virus; NBNC: non-B, non-C (hepatitis B virus surface antigen-negative/hepatitis C virus antibody-negative); ECOG-PS, Eastern Cooperative Oncology Group performance status; MTA, molecular target agents; AFP, alpha-fetoprotein; DCP, des-gamma-carboxy prothrombin; MVI, major venous invasion; BCLC, Barcelona clinic liver cancer; TNM stage, tumor node metastasis stage; LCSGJ 6th, the Liver Cancer Study Group of Japan, 6th edition;

Supplementary Table S2. Clinicopathological characteristics of 15 HCC cases with histopathologically confirmed necrosis in hepatectomy specimens

Variable	n=15
Median age, years	64 (50-83)
Sex (Male/Female), n (%)	13/2: 86.7/13.3%
Cause of HCC (HBV/HCV/NBNC), n	3/6/6: 20.0/40.0/40.0%
Child-Pugh score 5/6, n: %	9/6: 60.0/40.0%
ECOG-PS (0/1)	15/0: 100/0%
AFP (ng/mL)	11.0 (2.4-627.0)
DCP (AU/mL)	56 (17-82700)
Median tumor size (mm)	70.5 (54-86)
No. of tumor nodules (solitary/multiple)	11/4: 73.3/26.7%
Differentiation (well/moderate/poor)	0/0/15: 0/0/100.0%
MVI, n (%)	1 (6.7%)
BCLC stage (A/B/C)	2/12/1: 13.3/80.0/6.7%
TNM stage (II/III/IV) LCSGJ 6th	10/4/1: 66.7/26.7//6.7%

HCC, hepatocellular carcinoma; HBV, hepatitis B virus; HCV, hepatitis C virus; NBNC, non-B, non-C (hepatitis B virus surface antigen-negative/hepatitis C virus antibody-negative); ECOG-PS, Eastern Cooperative Oncology Group performance status; TKI, tyrosine kinase inhibitor; AFP, alpha-fetoprotein; DCP, des-gamma-carboxy prothrombin; MVI, major venous invasion; BCLC, Barcelona clinic liver cancer; TACE, transcatheter arterial chemoembolization; TNM stage, tumor node metastasis stage; LCSGJ 6th, the Liver Cancer Study Group of Japan, 6th edition

Supplementary Figure S1

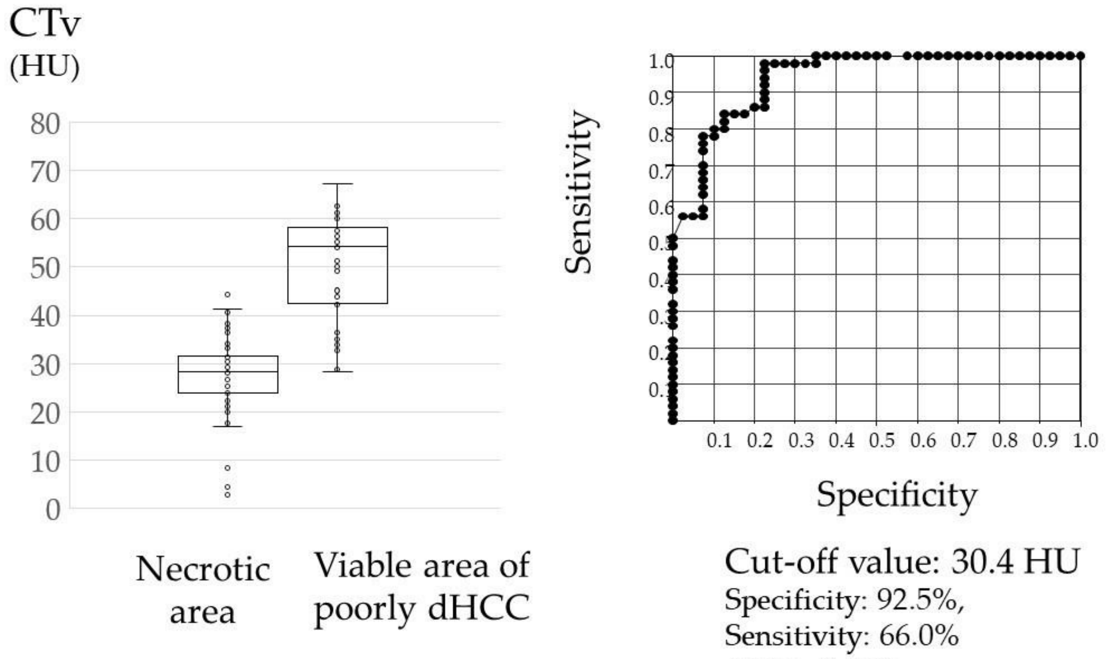

Cut-off value: 30.4 HU
Specificity: 92.5%,
Sensitivity: 66.0%
AUC = 0.939
(95% CI: 0.912-0.963)
